# EFL teachers’ motivational complexity and dynamics during collaborative action research

**DOI:** 10.3389/fpsyg.2022.957476

**Published:** 2022-07-15

**Authors:** Hongying Zheng, Tingting Huan

**Affiliations:** ^1^School of Foreign Languages, Sichuan Normal University, Chengdu, China; ^2^Faculty of Education, Sichuan Normal University, Chengdu, China

**Keywords:** EFL teachers’ motivation, collaborative action research, complexity, dynamics, perturbation

## Abstract

Collaborative action research (CAR) is a process that brings about changes to teachers in social situations as the result of collaboration. The adoption of complexity theory, accordingly enables the examination of teachers’ motivation during the CAR from a complex and dynamic perspective, while the inclusion of self-determination theory (SDT) highlights the trajectory of teachers’ self-development. Aiming to reveal EFL teachers’ motivational complexity and dynamics during the CAR, this study investigated six EFL teachers’ motivation by conducting semi-structured interviews, observations, and reflective writings. The results indicate that EFL teachers’ motivation in the context of the CAR is complex and dynamic, which cannot be simply divided into intrinsic and extrinsic motivation. The dynamic interaction between the teachers’ psychological self and the contexts further leads to different trajectories of motivational change. Moreover, as an important source of “perturbations” promoting the teachers’ motivational change, the CAR accumulates all the possible support for teachers to meet their psychological needs in terms of competence, autonomy, and relatedness. The findings provide insights into EFL teachers’ motivation and offer useful suggestions for teachers’ professional development.

## Introduction

*General Senior High School English Curriculum Standards (2017 version)* [MoE (The Ministry of Education of the People’s Republic of China), 2018] advocates a new concept of “competence-oriented teaching” in English as a foreign language (EFL) teaching in China. Such advocation poses great challenges to senior high school EFL teachers as they have to shift their teaching focus from “knowledge, skill, and affect” to “language ability, cultural awareness, thinking capacity, and learning ability,” which accordingly creates growing needs to learn to teach. In the process of teacher development, action research has been regarded as “a self-reflective, critical, and systematic approach to exploring one’s own teaching contexts” (Burns, 2010, p. 2). As a form of action research, collaborative action research (CAR) emphasizes cooperation between members and can be more effective in changing practice than reflection and discussion about practice alone (Vaino et al., 2013). However, the practical needs for teachers’ professional development do not naturally lead to teachers’ involvement in CAR because of many interfering factors such as teachers’ motivation (Zeynep Muğaloğlu and Doğança, 2009), school context (Glanz, 2016), the quality of action research approach (Webb et al., 2001) and so on. Among all these factors, teachers’ motivation is at the foundation of CAR cycle because it is practical, participative, tentative, and critical which can set teachers in the mood of anxiety (Bleicher, 2013).

Defined as “the marshaling of feelings of enthusiasm, zeal, and confidence” (Goleman, 1995, p. 79), motivation is “a cognitive phenomenon full of dynamics and complexity” (Dörnyei and Ushioda, 2011, p. 3). Previous studies on motivation, however, mainly focus on the classification of teachers’ motivation into extrinsic, intrinsic, and altruistic ones (Thomson et al., 2012), which ignores the features of motivation as “an ever-changing one that emerges from the processes of interaction of many agents, internal and external, in the ever-changing complex world” (Ellis and Larsen-Freeman, 2006, p. 563). As a key impetus for teachers’ participation in CAR, teachers’ motivation should be examined in a complex and dynamic background so that a holistic picture of teachers’ motivational change can be captured (Ryan, 2012; Edwards and Burns, 2016).

In the process of CAR, teachers interact in social situations as the result of collaboration. Moreover, teachers’ interactions in the macro-context of national educational reform, the exo-context of schools and collaborative communities, and the micro-context of classrooms, may highlight the complex and dynamic features of their change in motivation. However, individuals are, in most cases, not passive but active in integrating the interactions into their organizational cognitive structure. Therefore, in order to regain a better understanding of teachers’ motivation in the complex and dynamic contexts, the study delves into the teachers’ ***self*** to uncover the key motivational trajectories of teachers and explore the important influencing factors which take the role of attractors in the process of CAR.

## Understanding teachers’ motivation in complex and dynamic contexts

As a crucial factor related to teaching and learning, motivation has attracted many researchers’ interest, such as motivation to teach (Watt and Richardson, 2008), motivation to take training (Gorozidis and Papaioannou, 2014), and motivation to improve teaching (Schellenbach-Zell and Gräsel, 2010) as it “determines what attracts individuals to teaching, how long they remain in their initial teacher education courses and subsequently the teaching profession, and the extent to which they engage with their courses and the profession” (Sinclair, 2008, p. 80). Motivation has been studied in the field of education for over 60 years with the

focus mainly on students’ motivation, while the study of teachers’ motivation has only 20 years’ history with the focus mainly on teaching motivation (Al-Hoorie, 2018). The studies on motivation have been conducted from diversified theoretical perspectives, such as perspectives of social science, cognitive theory, and complexity theory (Boo et al., 2015; Dörnyei and Ryan, 2015; Han and Yin, 2016). In China, the study on motivation has been in a state of moderate development with the shift from theoretical research to empirical studies (Gao, 2012; Wang and Dai, 2015; Wang and Wang, 2019; Liu et al., 2020). The empirical studies focus on the exploration of teachers’ beliefs about motivation (You, 2010; Zhao and Wang, 2010; Wang et al., 2015), the relationship between learners’ motivation and their learning practice (Xu, 2014), teachers’ different motivational strategies (Yu, 2012; Wang and Dai, 2015; Yang and He, 2019), and so on. However, few studies have worked on teachers’ motivation in doing research (Liu, 2016), not to mention in collaborative action research.

As a way of improving teaching, collaborative action research (CAR) was rooted in action research, which was first established in the 1940s by Kurt Lewin aiming to improve professional practice by equipping teachers with the skills required to explore and meet the challenges in the educational context. Compared to action research, CAR puts more importance on “climates of inquiry in communities of practice” (Mitchell et al., 2009, p. 345). Hence, CAR enables teachers to take part in a meticulous self-appraisal of their current practice in reflective and reflexive processes in a community, which, accordingly, puts teachers under the pressure of challenging and criticizing themselves in a community of practice. Hopkins (1997, p. 2) referred to this as “a sense of anxiety and feelings of incompetence, associated with relearning and meaningful change.” Therefore, not all teachers are willing to get involved even though they know the benefits. Moreover, as motivation is fragile and context-related, such factors as stress, restricted autonomy, insufficient self-efficacy, lack of intellectual challenge and inadequate career structure, can be demotivating (Dörnyei and Ushioda, 2011). According to Dörnyei and Ushioda (2011, p. 3), motivation is a cognitive phenomenon full of dynamics and complexity which cannot make consensuses among researchers, but it certainly relates to the choice, the persistence, and the effort expended on a particular action. When teachers join CAR that may last for several months, the motivational process changes during different sub-phases in a complex way. Thus, it is necessary to understand teachers’ motivation in complex, dynamic, and system-interaction contexts.

**Figure 1:**
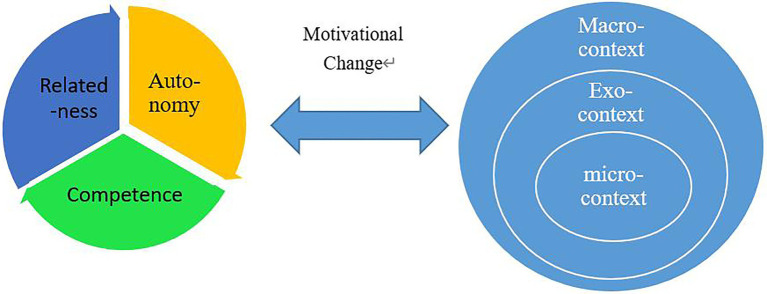
Teachers’ interaction with different contexts.

Originated from science, complexity theory believes that the cognition and behavior of individuals show a tendency of complex dynamics due to the interaction between human beings and the environment (Opfer et al., 2011). During the process of CAR, we conceptualized EFL teachers’ motivation as a complex, dynamic, contextualized open system that is neither reducible to discrete components nor amenable to simple linear equations of prediction. In this system, EFL teachers’ motivation is composed of different types of agents and elements, interacting with each other in specific contexts in China (see Figure 1). During the CAR process, teacher’s ***self*** is an agent interacting with different contextual factors, which contributes to the change of teachers’ motivation. That is, contextual factors, such as educational policies in the macro-context, school ethos and the community of CAR in the exo-context, and the classrooms in the micro-context interact with teachers, resulting in the complex and dynamic features of teachers’ motivation. By employing the framework of complexity theory, we can trace how participants adapt themselves in the process of interacting with dynamic environments (Holland, 2003), and these adaptive interactions—termed coordination dynamics—shed light on how functionally significant patterns of coordinated behavior emerge from initial states, persist in context, and adapt or change through time (Jirsa and Kelso, 2011). Moreover, it is predictable, from the perspective of complexity theory, that teachers’ motivation changes when teachers interact with different contexts. However, such motivational change may not be predictable as it relates to one’s psychological state. Therefore, the exploration of the trajectory of teachers’ motivational change in the process of CAR requires integration of complexity theory that focuses on teachers’ complex and dynamic contextual interactions as well as the exploration of teachers’ psychological process of being ***self*** during the process of CAR.

## Understanding self in the perspective of SDT

According to self-determination theory (SDT), there is no well-marked boundary between extrinsic and intrinsic motivation (Deci and Ryan, 1985). During the motivational internalization process, if extrinsic goals can be internalized to some extent, the extrinsic forms of motivation can be placed on a continuum representing different degrees of external control or internal regulation (self-determination; Dörnyei and Ushioda, 2011, p. 24). That is, extrinsic goals that are completely internalized in one’s self-concept can coexist with intrinsic regulation of motivation. While the task of ***self***, the core foundational concern in SDT, is to regulate inputs from external and internal contexts in an active integrative process (Ryan and Deci, 2019). SDT emphasizes people’s psychological growth, integration, learning and connection with others, and thus self-determination is highly related to people’s psychological needs for autonomy, competence, and relatedness (Deci and Ryan, 2020). **Autonomy** refers to a sense of initiative and ownership in one’s actions along with their outcomes. When people have the right to choose and make decisions, their sense of autonomy can be enhanced (Deci and Ryan, 2020), and they are more likely to internalize and integrate the value of behaviors (Reeve, 2009). **Competence** means the sense of mastery, the feeling of being qualified in reaching desired ends (Ingledew et al., 2004), a reflection of one’s sense of confidence and efficacy (Stone et al., 2009). People who feel a sense of competence are more likely to engage in challenging tasks (Deci and Ryan, 2002). Teachers’ sense of competence is regarded to relate to positive teaching behaviors, like enthusiasm for teaching (Skaalvik and Skaalvik, 2010). **Relatedness** concerns the feeling of belonging and connection (Kowal and Fortier, 1999). When a person is valued, respected, and accepted, the person can enhance the sense of relatedness (Niemiec and Ryan, 2009). Teachers’ sense of relatedness is reported to be important because of its impact on teachers’ motivation and well-being (Baard et al., 2004).

It is evident that SDT emphasizes people’s inherent motivational propensities for learning and growing, which has worked as a framework to analyze the process of people’s behavior and motivation in different fields (Nie et al., 2015; Deci et al., 2017; Standage and Ryan, 2019). Motivational processes, from the perspective of SDT, can also be more directly linked with specific teaching practices and curricula that elicit and scaffold learning in different subject areas (Ryan and Deci, 2019). Thus, we employ SDT, together with complexity theory, to gain a better understanding of how teachers’ motivation changes by dynamically balancing their psychological needs and their interaction with different social contexts in CAR process. In our framework, the perspective of complexity theory focuses on the teachers’ interaction with different contexts, while the lens of SDT pays more attention to the basic needs of teachers’ psychological states when they interact with contexts.

## Methodology

This is a qualitative case study that focuses on regaining an understanding of teachers’ motivation in collaborative action research. Based on the literature reviewed above, this study tried to understand teachers’ motivation in CAR by answering the following two research questions:

1. What are the trajectories of EFL teachers’ motivational change during the process of CAR?
2. What are the factors that contribute to the change of EFL teachers’ motivation in CAR?

### Research context and participants

With the rapid development of English language teaching and learning in China in recent years, EFL teachers’ professional development has gained momentum in promoting EFL teaching qualities. On the one hand, the new national English curriculum standards pose new challenges for EFL teachers, especially with the growing prominence of thinking competence in language learning (Chen et al., 2019; Zheng, 2020). On the other hand, *Professional Standards for teachers (Trial)* [MoE (The Ministry of Education of the People’s Republic of China), 2012] point out that it is essential for teachers to be equipped with the ability to do research in view of improving teaching practice. As the call for “teacher-researcher,” teachers should be and can be researchers to do research based on the reflective thinking from daily practice. At the operational level, publications and research projects are the must in assessing teachers’ professional titles. As teacher-researchers, we initiated a project called “Research on developing senior high school students’ thinking competence by visualizing thinking in English classroom” that aims to enable senior high school EFL teachers to develop students thinking competence in EFL classrooms.

Among the teachers who took part in the project, six of them volunteered to join the sub-project of CAR, which aims to figure out the ebbs and flows of teachers’ motivation during the process. As these teachers are volunteers, there is a great variety of the participants in terms of gender, age, schools, and years of teaching, which in a sense, provides rich contextual issues to the study (see Table 1). During the study, we played the role of facilitator, guide, formulator, summarizer of knowledge, and raiser of issues. Moreover, we invited a teacher-researcher officer, Mrs. Huang, to join in the research. Thus, we together played the role of etic observers examining EFL teachers’ motivation and influencing factors, which together with the emic perspective from the participant teachers, provides the research with both emic and etic perspectives.

**Table 1:** Profile of the participants.

Name	Gender	Age	Degree	Schools	Years of teaching
Han	Female	44	Bachelor	National key school A	22
Max	Male	41	Bachelor	National key school B	19
Linda	Female	25	Master	Private school C	2
Mary	Female	46	Bachelor	National key school D	25
Helen	Female	35	Bachelor	School E in a county	13
Jessica	Female	47	Bachelor	School F at the fringe of a city	27

### Research methods

In order to “develop an in-depth exploration of a central phenomenon,” this study adopts a case study to collect data “from multiple sources and perspectives” (Creswell, 2012, p. 206), which is believed to be a suitable method allowing researchers to “construct reality in interaction with their social worlds” (Merriam, 2009, p. 22). Data collection lasted for more than 3 months, during which all participants were involved in several cycles of action research until they got satisfactory results. According to Dörnyei and Ushioda (2011, p. 198) research on motivation is a formidable task for three reasons: motivation is abstract and not directly observable; motivation is a multidimensional construction; motivation is inconstant and dynamic. To ensure the validity of this research, interviews, observation, and reflective writing were adopted to collect data.

**Figure 2:**
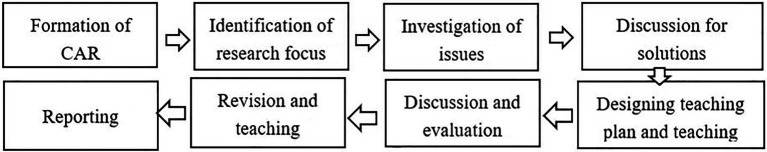
The CAR process.

CAR in the study created a community of practice for six teachers to develop their ways of teaching thinking in senior high school EFL classrooms. As these teachers were from different schools, they created a WeChat group for online discussions and they met occasionally offline to discuss the teaching plan. The final product of the CAR was a group presentation at a national teacher education conference after 2 months’ research. The CAR started in September 2020 with the researcher’s lecture on the topic of visualized teaching strategies in EFL teaching so naturally when the CAR was formed, the teachers identified the research focus on developing students’ thinking competence. They began their action research by choosing one unit to design and conduct teaching. By investigating their own teaching, they identified the questions they used in teaching as issues for research. With group discussions and literature readings, the teachers began to design their own teaching, and then they pooled their ideas together to discuss until they were satisfied. After another round of discussion and evaluation, they revised their teaching plan and put it into practice. Finally, they reported at the national conference in November 2020 with satisfactory results (see Figure 2). The whole process was collaborative as they gave each other suggestions and they pooled ideas together for a final public presentation.

During the process of CAR, every teacher was interviewed, observed, and asked to write reflections. Three times of formal interviews were conducted aiming to trace the psychological change of the teachers’ motivation and the influencing factors. The first interview was at the beginning of CAR. The second time was chosen at the time of the “critical moment” according to the teachers’ reflective teaching. The third time of the interview was conducted towards the end of CAR, asking for their own depiction of their motivational change. The semi-structured interview was adopted based on the questions related to autonomy, competence, and relatedness. For example, questions about autonomy include: (1) what made you join the CAR? (2) what do you think of your design? (3) what are the factors that encourage you to go on researching? Questions about competence are: (1) do you always feel confident in making the change? If not, what makes you think like that? (2) what will you usually do when you do not agree with others? Questions about relatedness include: (1) what kind of role do you think you play in the group? (2) do you feel a sense of belongings in the group? (3) what makes you continue in this research group? All these interviews were conducted in Chinese and were audio-recorded. The interview records were transcribed and thematically analyzed.

In this study, classroom observation was employed to record the teachers’ changes in their practices. Classroom observations were conducted three times on each teacher. The video recording was used to record teaching and was transcribed for further discussion. Moreover, as the study of the teachers’ motivation needs to delve into the teachers’ inner ***self,*** it is important and necessary to invite the teachers to reflect on their thoughts and practice. The reflective writing was used to record the teachers’ inner mind. During the cycles of CAR, participants wrote their personal reflections to record decisions that they had made, steps they had taken, the outcomes they had got, and the possible reasons for these. These reflections are considered as a source of data to triangulate with the interview, observation data for teachers’ motivational changes and possible influencing factors.

Content analysis was adopted to analyze all the data collected from interviews, observations, and reflective writings under the guidance of research questions. According to the research questions, two major coding frameworks were established to reveal the teachers’ motivational changes and the influencing factors. For example, in the exploration of the trajectories of the teachers’ motivational change, the coding schemes were first set from common knowledge of different stages of change: “Initial stage – changing stage – final stage.” After setting the sub-codes, different contents were analyzed to be put into different categories. After analyzing the features of the teachers’ motivation at each stage, the coding schemes were emergent as “accelerating,” “growing,” and “fluctuating” trajectories (see Table 2).

**Table 2:** A sample of the coding framework of the trajectory of teachers’ motivational change.

Research questions	Emerging coding schemes	Sub-codes	Initial codes (examples)
1. What are the trajectories of EFL teachers’ motivation during the process of CAR?	Accelerating trajectory	Initial stage	*… I am supposed to set an example … So, I come here. (LindaI1)*
		Changing stage	*… got very excited at the wish to change… (LindaI2)* *… will be very fruitful. (LindarI2)*
		Final stage	*… more and more engaged (Linda R4)*
	Growing trajectory	Initial stage	*I am the team leader in my school. It is my responsibility to … (MaryI1)*
		Changing stage	*… closely related… (Mary I2)* *… proud to be the team leader (Mary R5)*
		Final stage	*… felt belongings and competence (MaryI3)*
	Fluctuating trajectory	Initial stage	*I cannot keep pace with others if I do not keep learning (JessicaI1)*
		Changing stage	*I was so frustrated…doubt myself. (JessicaR3)* *… feel confident again (Jessica R4)* *… want to give up (JessicaR5)*
		Final stage	*… I have courage to go on … (JessicaI3)*

Moreover, the authors also referred to the template of “competence,” “autonomy,” and “relatedness” from self-determination theory in order to reveal the features of the teachers’ motivation at each stage. In analyzing the influencing factors, the coding scheme was set according to the theoretical framework of the study: the teachers’ psychological needs and the contextual factors. The analysis of social contexts was based on three levels of contexts: the macro-context, the exo-context, and the micro-context from complexity theory. After setting the sub-codes, similar process of putting contents into different categories was followed. For example, the teachers’ expressions like “the new curriculum reform,” “the requirement of teachers’ professional promotion” were put into the sub-code of macro-context. To ensure the reliability of data analysis, the inter-raters of the participant teachers and two researchers of Mrs. Zheng and Mrs. Huang were invited to analyze the data separately for the first teacher’s data for agreement.

## Findings and discussion

The study aims to reveal the trajectories of the teachers’ motivational change and the influencing factors that contribute to the change with the data collected from interviews, observations, and reflective writings.

### The trajectories of EFL teachers’ motivational change

The study on the trajectories of the teachers’ motivational change followed a timeline of “Initial state-critical moments-final state,” which recorded the teachers’ motivational complexity and dynamics from the teachers’ psychological self and their interaction with the contexts.

#### EFL teachers’ varied initial motivation

Six senior high school EFL teachers held varied motivations when joining the CAR. The first interview revealed that the teachers’ (Han, Max, Linda, Mary, Helen, Jessica) initial motivation in joining the CAR varies. Linda was motivated by the satisfaction of her needs for competence, Mary and Han were motivated by the satisfaction of her needs for relatedness, and Helen, Jessica and Max were motivated by their strong autonomy in learning for a change.

Among the six teachers, Linda was tagged with a high degree of professional identity as she was the only one with a master’s degree majoring in English curriculum and teaching. When I asked why she wanted to join us in the first interview, she said with an embarrassed smile,

Extract 1

*As you know, I am a new teacher in my school and I feel so far so good. But my mentor*^1^ *told me that I was supposed to set an example for other teachers in doing research. So I come here (LindaI1)*.^2^

Obviously, Linda joined the CAR group at the suggestion of her mentor, which indicated Linda’s extrinsic motivation. However, when we traced down to her inner self, more information about her psychological state was revealed. Due to the study experience of her master’s degree, she was confident and open-minded to join such research “to learn something.” Therefore, Linda’s initial motivation was triggered by the satisfaction of her need for competence.

Unlike Linda, Mary joined the group because of her desire for professional development as a team leader in her school. Even though Mary has been teaching in her school for 25 years, she felt an urgent need to learn new concepts in the national English curriculum standards so that she can work better as a team leader.

Extract 2

*You know I’ve been an English teacher for 25 years since I left college. Now, I am the team leader in my school. It is my responsibility to learn those new concepts and share my ideas with other teachers in my team. And I think it will be good to join such a group with the company of teacher-researchers and teachers from other schools. It is an opportunity to communicate with others (MaryI1).*

When reading between the lines, we can see with 25 years of teaching experience, Mary lost her passion for any change as she had experienced several rounds of teaching reform.

Extract 3

*To be frank, we teachers are tired of so many rounds of educational reform with some so-called new concepts. After the noise and bustle in the classroom, what we teachers do is still the same if there is no change in the final examination. But when the new round of teaching reform comes, I still join in the research to fulfill my duty as an English teacher and the team leader (MaryI1).*

For Mary, she was motivated by autonomy for improvement and her identity as a team leader. Similarly, Han was motivated by her identity as a team leader as she wanted to perform her role well in her school.

Max approached us with questions, and his motivation was “to find answers.” Max, a senior teacher in a key middle school with almost 20 years of teaching experience, was interested in developing students’ thinking ability in English classes. After the release of the new curriculum standards, he tried by himself to improve his teaching practice and even published an article about teaching thinking. However, he still came to us with questions on cultivating thinking competence in his daily EFL teaching. He said,

Extract 4

*The new English curriculum standards promote a brand-new concept of cultivating students’ thinking competence. I figured out there were many problems in my teaching, and I tried several times to improve my teaching practice. However, I am still looking for proper teaching approaches. I want to find a set of scientific and systematic ways of teaching (MaxI1).*

Although Helen and Jessie joined the CAR autonomously, the impetus was different in that Helen was pressured by the promotion of her professional titles and Jessie met the bottleneck in her career. In the study, the teachers’ motivation at the initial stage was revealed to be varied, which sets up different starting points for their different motivational trajectories.

#### EFL teachers’ motivational dynamics

EFL teachers’ motivational dynamics were also evident during the process of CAR when they interact with different contextual factors. The CAR provided an opportunity for the teachers to break their balance in teaching with either active or passive motivation for change. When the teachers joined the group, they demonstrated very differently towards the contextual influences in terms of their motivational change.

Take Mary for example, she joined the CAR because of her responsibility of being a team leader in her school. Compared with others, she was relatively low in motivation due to her belief that the educational reform will not bring about changes to teachers “if there is no change in the final exam.” In order to engage her into the team, the researchers nominated her as the group leader in the CAR, taking charge of organizing and arranging their activities. Although she was a bit reluctant to be the leader, she performed well gradually when we gave her more tasks to do as a team leader. In the second interview after a month’s time, Mary expressed her strong sense of belongings to this CAR group as she “was closely related to the group and should take the responsibility to do my work well” (MaryI2).

Interestingly, the first critical moment of their motivational change for most of the teachers happened during the process when they first examined their own classroom teaching with the focus on their questions in class.

Linda was very confident in her own professional knowledge according to her first interview. However, when she first presented her design for discussion, she was frustrated but then got very excited at the wish to change in the CAR. In one of her reflective writings, she wrote,

Extract 5

*After presenting my teaching design to the group which I was very confident, I was very frustrated when I found there were so many problems in my teaching. I did not realize that my questions failed to develop my students’ thinking competence. What I did was just the routines that have been regarded as the standard format for teaching by us teachers. I am so lucky to join the CAR to improve my teaching. I believe my experience here in the CAR will be very fruitful (LindarI2).*

For Linda, her motivation was lifted as she demonstrated a sense of initiative at this moment. After that, she participated in the CAR more attentively than before in that she constantly made the changes to her teaching design. She asked other teachers to give her suggestions. For many times, she even overthrew her whole design for brand-new ideas, during which process she became “more and more engaged” and autonomous. The following table (see Table 3) shows Linda’s revisions on the lead-in section of *A love of gardening* in Unit 6 Book 1 of the Senior high English textbook (Chen, 2019).

**Table 3:** Three versions of Linda’s lead-in questions.

	Original version	The third version	The final version
Lead-in questions	What can we learn from it? What will be talked about in the passage?	What is it in the picture? What do people usually do in their gardens? How do people living in it feel? According to the title, which questions will be answered?	What can you see in the picture? Do you want to live there? If you live there, how will you feel? How do people living there feel?

In EFL classroom teaching, teachers’ questioning, as an important part of teachers’ talk, can help develop students’ thinking, promote their activity engagement, and enhance the interaction between the teacher and students. In this case, question sequences were regarded as an effective tool to guide students to think more logically. The questions in Linda’s first version were very broad, which was not effective to promote students’ thinking. In the second version, Linda used the question sequence of “action – feeling” to guide students’ thinking. The final version upgraded the promoting effects of questions in that it started from students’ own actions to their feelings and further transferred the students’ own experiences to the understanding of other people. Linda was very satisfied with this change as she said “finally, I know how to use questions to guide students to think in the right direction advocated in the new curriculum” (LindaR5).

However, the same thing did not happen to Jessica. The change of Jessica’s motivation accompanies the adjustment of her state in the interaction with contexts. In the initial stage, Jessica joined the CAR with strong motivation to change. In her own words, “I cannot keep pace with others if I do not keep learning.” She thought it would be necessary for teachers to get involved in lifelong learning. However, despite the strong initial motivation, her CAR process did not go smoothly. One of her reflective writings is as follows:

Extract 6

*I am so frustrated. I think I have tried my best but it turned out that what I did was not even close to the standard. I am an experienced English teacher for more than 27 years and I am always confident. But this time, I begin to doubt myself. Actually, I am old enough not to push myself so hard. Why am I doing this? Should I just quit? Anyhow, it is not necessary for me to do such things (JessicaR3).*

Mrs. Huang and I noticed her frustration due to her reluctance to remodify her teaching design for the third time. We, together with the other teachers, analyzed her teaching plan and class videos and then gave suggestions to her teaching plan both online and offline for several times. After rounds of discussion, she finally succeeded in presenting a satisfactory design, which made her regain her confidence. During the whole process, her motivation ebbed and flowed many times. In her final interview, she said,

Extract 7

*I am so thankful to my dear teammates. I would have dropped out without your encouragement. Now I am reluctant to leave this group as I know I have the courage to go on with you guys around (JessicaI3).*

Max was the one who built his confidence in the CAR. Max was very silent when he first got into the group. But during the discussion, his ideas always attracted the other teachers’ attention and got accepted. Because of “peer recognition,” Max became highly motivated either in discussion or in taking up more work to do. It was evident that Max’s motivation changed when others’ behavioral responses changed. In the end, Max unanimously agreed to design the big question sequences of the unit, which has become a highlight of the final presentation. He created “PIRS” (Phenomenon-Influences-Reasons-Solutions) question sequences in teaching, raising questions including “What are the hard living conditions that people are faced with? What are the influences that nature has on human beings? What caused the trouble to human beings? What did they do to overcome the difficulties?” (MaxO3) These questions linked the whole unit together around the theme of “achieving one with nature,” which was highly acknowledged by the other teachers.

#### EFL teachers’ motivational balance

The CAR lasted 2 months with a final group presentation at a national conference as the ending point. Despite the fluctuation of the teachers’ motivation, the teachers achieved the unanimous enhancement towards the last offline group meeting 1 week before the presentation. The task of the last offline group meeting was to discuss the result of their last round of teaching based on their final version of their teaching design. Before the meeting, all the teachers sent their recordings of the last teaching to the group and were studied and evaluated. This meeting was regarded as monumental since all the teachers used the word “confident” in their reflective writings. In the researchers’ field notes, the teachers were depicted as “fully motivated,” and “with the same goal of performing well in the conference.”

In Max’s final reflective writing, he depicted his motivational journey as follows:

Extract 8

*[…]When Mrs. Huang told me that I was chosen to join the CAR, I felt very happy as I knew this will be a good opportunity for my growth. […]*

*[…]After the first online meeting when I got the task to identify issues in my teaching, I transcribed my teaching and examined it word by word with my colleagues. […]After the third online meeting, I gradually understood the ways of visualized teaching.*

*[…]The third offline meeting left me with a very deep impression as my teaching design was acknowledged by Mrs. Zheng.*

*[…]I got clearer and clearer about my ideas in teaching. With the presentation of “PIRS,” my work was acknowledged by all the other teachers. I felt so engaged and fruitful.*

*[…]After this round of revision (the last group discussion), I felt very confident about our teaching design and I believe we will do well at the conference (MaxR5)*

From Max’s reflective writing, it was evident that Max’s motivation accelerated with other people’s acknowledgement including his colleagues, the group members in the CAR, and the researchers until he felt confident.

For Jessica, she demonstrated a state of ups and downs with respect to motivation. But only in the last group meeting, she said in her reflective writing “…I was no longer hesitating. I had made 10 times’ revision and now I think I can do it in the final presentation.” (JessicaR5).

For Han, her motivational balance was achieved when she saw her students’ achievements in her last round of teaching. In her reflective writing, she stated,

Extract 9

*[…]In real teaching practice, the students met a lot of difficulties when I integrated the teaching of language points in relation to the elaboration of the thematic meanings of the unit. Then I knew I did it wrong. […] Now I used question sequences to lead my teaching reading and presented these sequences to the students for their output in their oral presentation. When I saw they can use them in their own words, I felt so fruitful. […]I became more and more confident in developing students’ thinking competence (HanR5).*

The above findings revealed the complex and dynamic features of the teachers’ motivation in the process of CAR. All the teachers’ motivational trajectories were different because of the interactive dynamics with different contexts. It is necessary, accordingly, to study how the teachers interacted with the contextual factors in the process of CAR.

### The factors influencing teachers’ motivational process

By studying the factors influencing the teachers’ motivational process, we infer that “perturbations” for teachers’ motivational change seemed to be caused by the interaction between the teachers’ psychological self and the contexts. Thus, we focus on the exploration of the teachers’ psychological ***self*** and the external systems in this section. Our findings of the teacher’s psychological self were from the teachers’ autonomy, competence, and relatedness. The contexts explored include the macro-context of the national educational policies, the exo-context of the school ethos and the community of CAR, and the micro-context of classrooms.

#### EFL teachers’ psychological self

During the 2 months period, all six teachers’ motivation was promoted with different trajectories. The above discussion on the teachers’ motivational complexity and dynamics revealed changes in the teachers’ psychological ***self*** regarding motivation. In this section, EFL teachers’ psychological ***self*** was elaborated according to the framework of SDT based on three psychological needs: competence, autonomy, and relatedness.

When teachers’ needs for competence are met, they will have a stronger sense of self-efficacy with themselves and a stronger impetus to take action. They are likely to get an ideal result in a challenging situation. Based on the data collected, the satisfaction of one’s competence for each teacher came from different sources. For example, Han joined the CAR with strong motivation. When she expressed her willingness to join the research, she said “This is not the first time I conduct action research, and I have a good understanding of how it works. I believe I can complete this study well” (HanI1). During the process, Han acted as one of the core members in our team and always showed support for the others. Conversely, when the teachers doubted their own competence, their motivation to participate dropped greatly. Jessica was a case in point. When she found that there was a deviation in her understanding of visualized thinking and the result of her first round of action research was not good, she wanted to give up. Luckily, with others’ support, she regained her self-efficacy during the process of CAR. In her final reflective writing, she wrote,

Extract 10

*In fact, English teachers have a lot of opportunities to do action research and they have the competence to conduct such research. If every teacher consciously implements AR into daily teaching and makes the research a norm, every teacher will be a teacher-researcher, which is of great benefit to both students and teachers themselves (JessicaR4).*

As revealed in “EFL Teachers’ Varied Initial Motivation”, Linda was motivated because of her competence in her professional knowledge, while Max’s motivation was promoted as his competence in developing students’ thinking skills had been constantly acknowledged by others. Moreover, Mary was motivated because of her competence as a team leader.

In terms of autonomy, it was evident that all participants had endorsed the value of CAR as a way of improving teaching practice and they experienced the sense of autonomy during the process. The teachers gained autonomy from different sources, such as the autonomy to join the CAR, to improve their teaching, to choose points for discussion and so on. During the whole process, as researchers, Mrs. Zheng and Mrs. Huang just stood aside to offer help if needed. However, such autonomy provided by the CAR had been gradually appreciated by the teachers. Helen was from a remote school, which was almost 2 h’ drive from the city where the other teachers work. At the beginning, she was not used to the autonomy the group provided. Every time, she came with questions and was eager to get the solutions right away from the researchers. However, when she was told that she needs to find out the answers by herself, she felt frustrated. Then she began to ask all the other teachers for solutions through WeChat. When the researchers found this tendency, we created an opportunity to encourage her to find out answers by herself in one of our offline discussions. This episode was recorded by Helen in one of her reflective writings.

Extract 11

*[…] although I am very confident in my school, here, together with all the excellent expert teachers in the group, I felt very nervous and I am afraid to make mistakes. However, Mrs. Zheng encouraged me so much. Today, when I got stuck in how to help students come up with the working principles of the Longji Rice Terraces, Mrs. Zheng stopped others to give me answers directly. When I took my time to think by using what I have learned in the past several weeks, I finally succeeded to design a task by linking what the students have learned to what they needed to produce. When Mrs. Zheng and my dear group applauded for me, I enjoyed it so much (HelenR3).*

It was true that Helen gradually enjoyed the autonomy the CAR provided to her and became more and more confident. During the CAR, different choices, explanations, and suggestions instead of rules were provided to support autonomy. As Han remarked in an interview, “I feel very relaxed here in the CAR. I am not afraid of making mistakes because I know there is a team that backs me up.” (HanI3)

The CAR provides teachers with the sense of belonging and support. In the CAR group, Mary was the nominated leader to organize meetings, while there was another self-elected team leader Max. He was the one whose ideas were always approved by the others so he gradually became the idea provider in the group. As mentioned above, the sense of belongings and acknowledgment by the group members were the source of relatedness for Max and Mary to achieve high motivation. When the presentation day came closer, all the teachers’ sense of belonging was growing. That was also the time when the teachers achieved the balance in motivation.

#### Contextual factors influencing the teachers’ motivation

When exploring the teachers’ motivation, it is unavoidable to probe how it was influenced by the contextual factors. The contextual factors acted as “perturbation” that motivates the teachers to change. In terms of the macro-context, the launch of the national English Curriculum Standards worked as the “perturbation” that caused the teachers to think about change and adaptation. When the teachers mentioned this educational reform, they all expressed the uneasiness about the application of the new concepts to their teaching. The teachers used such words to describe the educational reform as “pressure,” “a call for changes,” “a must that teachers should adapt to,” and so on. This “pressure” worked perfectly as an extrinsic force to urge the teachers to change. Moreover, some of the teachers (such as Helen, Linda and Max) mentioned that research skills were very essential for their promotion of professional titles. Especially, Helen regarded it as one of the most important motivations to join the CAR. Strangely, no one mentioned this during the whole process until the last meeting when Max raised the issue of publication. He asked, “is it possible for us to publish our research as the promotion of our professional title needs this?” His question was echoed by Linda as she was also worrying about “getting promoted professionally.”

As for the exo-context, the initiation of the CAR met the teachers’ psychological needs (which vary) so as to motivate the teachers to a certain degree. All the activities and people in this community were all important influencing factors to the teachers. For example, the acknowledgements from the researchers met the teachers’ needs for competence; the encouragement from the group members met the teachers’ needs of relatedness, and the ways of working in the CAR met the teachers’ needs of autonomy. To different teachers, these factors, however, exerted different forces according to their different psychological needs. For example, the problems examined in the discussion were the forces for Linda to challenge and improve herself. But for Jessica, these problems made her frustrated. Meanwhile, teaching feedback from colleagues in the teachers’ own schools also exerted forces to motivate them. To Mary, the position of a team leader in her school motivated her to improve herself so that she can continue to be a model.

With respect to the micro-context, the students in the teachers’ own class were the strongest force in promoting the teachers to change. The students’ positive feedback and their improvement in EFL learning were the most frequently mentioned “perturbation” that enabled the teachers to change. As Han wrote in her reflective writing:

Extract 12

*Having been a teacher for so many years, I used to be very satisfied with my teaching. However, I felt a little bit uneasy facing my students after taking part in this research. By learning and reflecting, I noticed the absence of developing students’ thinking skills in my class for those years (HanR1).*

After she realized her lack of competence in helping students develop thinking skills in EFL teaching, Han was even more motivated to get involved in the CAR. The distinctive change happened right after her first reflective writing. She began to initiate questions and answers in the online discussion platform. And she was the first one to try out her new ideas in teaching (see Table 4).

**Table 4:** Han’s question sequences in teaching unit 6 *at one with nature* (ZhengO1).

Thinking levels	Question sequences
Understanding	1. Why can they (Longji Rice Terraces) attract so many visitors?
	2. Who built the terraces? When did the work begin and how long did it last?
	3. What problems did the local people solve by working with nature?
	4. Why is it significant to build these terraces? How is it designed?
	5. Why do the local people still keep their traditional way of growing rice?
	6. What’s the author’s main purpose of writing this passage?
Applying	In what other ways did people live in harmony with nature? Give examples.
Transferring	If you have a chance to visit Longji Rice Terraces, what should you do to be an eco-tourist?

Han designed her questions based on the activity-based approach to English learning advocated in the new curriculum standards from three levels: learning and understanding, applying and practicing, and transferring and innovating. When her teaching helped students understand the meaning of the text better, Han felt rewarded in doing the research in the CAR. When she doubted about the effectiveness of her questions that are intended to help students transfer what they have learned to real life, she was even more motivated to find answers to the questions in the follow-up discussion in the CAR.

### Discussion: teachers’ motivational complexity and dynamics

The purpose of this study is to gain a better understanding of the motivational process in CAR with the framework of SDT and complexity theory. The study reveals the teachers’ motivational complexity and dynamics in the CAR by analyzing the data from interviews, observations, and reflective writings. Firstly, teachers’ motivation system is complex, which cannot be simply divided into intrinsic and extrinsic motivation. The teachers’ motivational complexity is evident in its varied initial states, such as Linda’s motivation urged by her mentor, Mary’s motivation for being a good team leader, Helen’s motivation for professional promotion, and Max’s motivation for finding answers to his puzzles. With different initial states, the teachers began to interact with different contextual factors, aiming to meet their changing psychological needs, which leads to a variety of motivations. For example, Linda joined the CAR due to some forms of extrinsic motivation such as “the suggestion of a mentor.” In the meantime, she mentioned her competence in “doing the research.” And at the last interview, she emphasized her needs to “develop professionally,” “publish more papers” and “get a professional title.” As Sinclair (2008) pointed out, mixed motivation seems to have a more positive long-term effect and can overcome possible clashes with reality. It is true for Jessica when her motivation only related to her competence, she constantly felt frustrated. As her psychological needs became varied when she was getting involved in the CAR, the acknowledgements from others and the achievements her students made had helped her overcome a “possible clash with reality.” Thus, the multiplication of the teachers’ psychological needs, which are perturbative to the teachers’ motivation, can help teachers achieve psychological satisfaction during the change.

Secondly, the dynamic interaction with the contexts further leads to different trajectories of motivational change. There are three major types of motivational trajectories: accelerating motivation from low to high with a sharp turning point, growing motivation with a moderate increasing speed, and fluctuating motivation with ups and downs during the process of CAR. For Linda, she joined the CAR with moderate motivation for improving her competence in doing research. During the process, the turning point was the moment she found challenges, which further motivated her to be better until she achieved a stable state of motivation. Her motivational trajectory can be regarded as an accelerating motivation with the need of competence satisfied. For Mary and Max, their motivational changes were accompanied by the gradual acknowledgements of their leading roles in the CAR. Thus, their motivational trajectory can be labeled as growing motivation with the need of relatedness satisfied. For Jessica, her strong motivation decreased when she was first beaten by the failure in providing a satisfactory teaching design. With the help and encouragement from the teammates, she regained her confidence. However, with the second failure in the research, she returned to the state with decreased motivation. The further acknowledgement from the researcher and the achievements made by the students pulled her up to continue until she finally succeeded in the presentation. Such fluctuating motivation was due to her incompetence in the research. Such complexity and dynamics of the teachers’ motivation confirmed the tenet of SDT: external and internal motivation is not binary but a continuum (e.g., Ryan, 2012).

Thirdly, it is safe to say “perturbation” is an important influencing factor to motivate teachers to reflect and change. “Perturbation” from different contexts may lead to dissonance in some teachers’ cognition and become a driving force to change. But for some other teachers, constant and purposeful “perturbation” from others will help them strengthen and internalize motivation. Apart from different personal contexts of the teachers, the satisfaction of people’s basic psychological needs can illustrate this difference. In general, the satisfaction of one’s basic psychological needs has proved to be related to the autonomous forms of motivation (Standage and Gillison, 2007), and these interventions which supported autonomous motivation were believed to increase these basic needs (Edmunds et al., 2007). In our study, the nonlinear motivational change of each participant is related to the satisfaction of their needs, like Han’s competence need, Linda’s relatedness need. But we should keep in mind that interventions that provide synergistic support for these needs tend to get a greater behavioral engagement than support for each need alone (Deci et al., 1994). Take Jessica for example, when her need for competence was unsatisfied, our team provided all-around support which made her feel “related,” “capable” and “not afraid to do any research.”

As stated above, the contextual factors always work as “perturbation” to initiate changes in the teachers’ motivation. However, it is also evident in the study that the interaction between the teachers’ psychological needs and contextual factors enables teachers to adapt to the CAR. In the study, when Jessica’s competence was challenged, her motivation decreased. But her interaction with the context, including the acknowledgement from the researchers and the encouragement from the group members helped her to regain her confidence. Even though the teachers experienced different trajectories of motivational change, they managed to adapt themselves to the research and did a great job in the conference group presentation. Therefore, the teachers demonstrate adaptive motivation, which sheds light on how the patterns of the teachers’ motivational change emerge from initial states, fluctuate in contexts, and achieve a balance in the end. Such finding echoes Jirsa and Kelso (2011) research on coordination dynamics in understanding complex systems. The concern about the teachers’ psychological needs and how they adapt themselves to the contexts help to find out the patterns of motivational change. More direct support, accordingly, can be given to teachers when they struggle with “perturbation” at the time of educational reform and change.

## Conclusion

The teachers in the CAR experienced different trajectories with the dynamic interaction between the teachers’ psychological needs and contextual factors. The CAR, as the perturbation to promote the teachers’ motivational change, is proved to be helpful for teachers’ motivational growth. Moreover, the CAR accumulates all the possible support for teachers to meet their psychological needs in terms of competence, autonomy, and relatedness. In this way, the CAR can create a community of practice for teachers to develop professionally and psychologically.

In the meantime, the teachers’ motivation change in the process of CAR is complex and dynamic, which may be regarded as unpredictable. However, the current research finds out the patterns of motivational change by exploring how the teachers’ psychological needs adapt to the contexts. In this case, when the teachers are faced with challenges in teaching, the CAR can be suggested as an effective way to enhance teachers’ motivation of adaptation and change. And the exploration of the teachers’ psychological needs, the complexity of their motivation, and the dynamic interaction between their psychological needs and the contexts can help create a motivation-favored environment. Therefore, although the CAR is time-consuming and required coordinated efforts, it is worthwhile for teachers and researchers to try in the time of educational reform.

## Data availability statement

The original contributions presented in the study are included in the article/supplementary material, further inquiries can be directed to the corresponding author.

## Author contributions

HZ and TH contributed to the research and paper writing. HZ conceptualized the study, collected the data, and contributed to the subsequent revisions. TH analyzed the data and wrote the first draft. All authors contributed to the article and approved the submitted version.

## Funding

This article was supported by The educational research project of “fourteenth Five-year plan” –China Basic Foreign Language Education Research & Training Center (Grant number: QJZX2021020416).

## Conflict of interest

The authors declare that the research was conducted in the absence of any commercial or financial relationships that could be construed as a potential conflict of interest.

## Publisher’s note

All claims expressed in this article are solely those of the authors and do not necessarily represent those of their affiliated organizations, or those of the publisher, the editors and the reviewers. Any product that may be evaluated in this article, or claim that may be made by its manufacturer, is not guaranteed or endorsed by the publisher.

## Footnotes

^1^In order to help new teachers to adapt to teaching career, some schools in China will arrange senior teachers as mentors to new teachers.

^2^LindaI1: Linda = participant; I=Interview; 1 = the first time of interview. In the following citation, similar note is applied as putting participants’ name in the first place, types of data in the second place and the times of collecting data.
